# The Editor's Letter-Box

**Published:** 1921-02-26

**Authors:** 


*To the Editor of* The Hospital.

Sir,—In a letter in the current issue of The Hospital signed Sister Caroline, the writer asks if the rank-and-file members of the College of Nursing are not entitled to know the policy and intentions of the candidates nominated for election to the College Council. Undoubtedly we are entitled to do so, but it is up to us to give candidates clearly to understand that we expect a clear statement from them as to their views on matters of vital importance to the well-being of the nursing services before the elections take place, and to invite them to visit any Centre and address the members if they desire to secure the votes of the members of that Centre. It is useless to call upon the College Council to pursue a forward policy unless we give them a practical assurance that we are prepared to support that policy, and if need be to subserve personal to professional gain. The strength of a union depends largely upon the single-mindedness and the far-sightedness of individual members of the union, as it is they who elect their leaders, and there is no reason why a professional union should not be as powerful as a trades union. The present plight of the nursing services is due to their lack of organisation in the past; it is for them now to heal the disunion in their ranks, to affiliate and amalgamate with other professional bodies, to form a political union, and to use the power so obtained as a lever to improve the conditions under which they work. It is the lethargy and the "Ca' Canny" policy of individual members of the nursing services which constitutes the gravest danger in the nursing world to-day.—Yours faithfully,

